# The multifaceted role of vitreous hyalocytes: Orchestrating inflammation, angiomodulation and erythrophagocytosis in proliferative diabetic retinopathy

**DOI:** 10.1186/s12974-024-03291-5

**Published:** 2024-11-14

**Authors:** Stefaniya K. Boneva, Julian Wolf, Malte Jung, Gabriele Prinz, Toco Y. P. Chui, Jacqueline Jauch, Anne Drougard, J. Andrew Pospisilik, Anja Schlecht, Felicitas Bucher, Richard B. Rosen, Hansjürgen Agostini, Günther Schlunck, Clemens A. K. Lange

**Affiliations:** 1 Eye Center, Medical Center, Faculty of Medicine, University Medical Center Freiburg, Freiburg, Germany; 2 Department of Ophthalmology, New York Eye and Ear Infirmary of Mount Sinai, Icahn School of Medicine at Mount Sinai, New York, NY USA; 3 Department of Epigenetics, Van Andel Research Institute, Grand Rapids, MI USA; 4 Department of Epigenetics, Max Planck Institute of Immunobiology and Epigenetics, Freiburg, Germany; 5 Institute for Anatomy and Cell Biology, Julius Maximilians University Würzburg, Würzburg, Germany; 6 Institute of Neuroanatomy, University Medical Center Hamburg-Eppendorf, Hamburg, Germany; 7 Department of Ophthalmology, St. Franziskus Hospital, Münster, Germany

**Keywords:** Hyalocytes, Vitreous macrophages, Proliferative diabetic retinopathy, Inflammation, Angiomodulation, Erythrophagocytosis, RNA-Sequencing

## Abstract

**Background:**

Despite great advances in proliferative diabetic retinopathy (PDR) therapy over the last decades, one third of treated patients continue to lose vision. While resident vitreous macrophages called hyalocytes have been implicated in the pathophysiology of vitreoretinal proliferative disease previously, little is known about their exact role in PDR. In this study, we address molecular and cellular alterations in the vitreous of PDR patients as a means towards assessing the potential contribution of hyalocytes to disease pathogenesis.

**Results:**

A total of 55 patients were included in this study encompassing RNA-Sequencing analysis of hyalocytes isolated from the vitreous of PDR and control patients, multiplex immunoassay and ELISA analyses of vitreous samples from PDR and control patients, as well as isolation and immunohistochemical staining of cultured porcine hyalocytes. Transcriptional analysis revealed an enhanced inflammatory response of hyalocytes contributing to the cytokine pool within the vitreous of PDR patients by expressing interleukin-6, among others. Further, increased angiopoietin-2 expression indicated that hyalocytes from PDR patients undergo a proangiogenic shift and may thus mediate the formation of retinal neovascularizations, the hallmark of PDR. Finally, RNA-Sequencing revealed an upregulation of factors known from hemoglobin catabolism in hyalocytes from PDR patients. By immunohistochemistry, cultured porcine hyalocytes exposed to red blood cells were shown to engulf and phagocytose these, which reveals hyalocytes’ potential to dispose of erythrocytes. Thus, our data suggest a potential role for vitreous macrophages in erythrophagocytosis and, thereby, clearance of vitreous hemorrhage, a severe complication of PDR.

**Conclusion:**

Our results strongly indicate a critical role for vitreous hyalocytes in key pathophysiological processes of proliferative diabetic retinopathy: inflammation, angiomodulation and erythrophagocytosis. Immunomodulation of hyalocytes may thus prove an essential novel therapeutic approach in diabetic vitreoretinal disease.

**Supplementary Information:**

The online version contains supplementary material available at 10.1186/s12974-024-03291-5.

## Graphical Abstract

In the left-hand side panel, a human eye of a patient with proliferative diabetic retinopathy with retinal neovascularizations (RNV) growing in the preretinal vitreous is schemed. Hyalocytes, the macrophages of the vitreous, are located mainly in the posterior part of the eye, close to the vitreo-retinal interface and, thus, RNV. Diabetic hyalocytes (on the right) modulate angiogenesis by expressing factors such as interleukin-6 (IL-6) and angiopoietn-2 (ANGPT2), contribute to fibrosis by transdifferentiation in myofiboblasts secreting extracellular matrix (ECM) components, and participate in erythrophagocytosis, as shown in this study

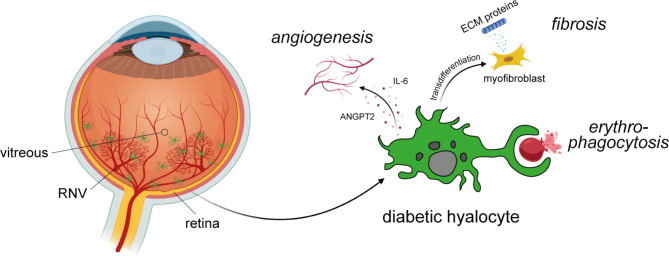

## Background

Diabetic retinopathy (DR) is a leading cause of blindness worldwide, with a global prevalence estimated to 93 million people in 2012 [1] projected to nearly triple in the USA by 2050 [2]. Proliferative DR (PDR), the advanced stage of the disease, is marked by areas of reduced retinal perfusion that generate an uncontrolled release of proangiogenic growth factors eventually leading to the formation of vulnerable new blood vessels called “retinal neovascularizations” (RNV, [3]). The compensatory attempts at revascularization of the ischemic retina generally result in unfavorable angiogenesis towards vitreous [4], the stimulus for this pathological preretinal formation of contractile vascular membranes remaining the matter of debate. Evidentially, a major driver of neovascularization in PDR is the vascular endothelial growth factor (VEGF) targeted effectively in routine treatment of diabetic macular edema (DME) and PDR by the application of anti-VEGF antibodies or decoy antibody receptors [5]. More recently, faricimab, a bispecific antibody acting as an inhibitor of both VEGF and angiopoietin-2 (ANGPT2), was introduced to enhance treatment for neovascular eye disease [6]. Those successes notwithstanding, other pathways likely contribute to PDR pathogenesis, as the disease often progresses even under continuous anti-VEGF therapy [7] and addition of ANGPT2 inhibition has improved outcomes in only a minority of DME patients switched from aflibercept [8]. A deeper understanding of additional mechanisms underlying PDR development is therefore critical for unveiling alternative therapeutic options, given the disease’s growing socio-economic impact.

Clinical evidence clearly demonstrates that the vitreous is critically involved in PDR, as florid preretinal neovascularization in PDR is mitigated following posterior vitreous detachment (PVD, [9]) and generally does not recur post-vitrectomy [10]. It appears likely that additional components of the vitreous, other than the supporting structure of vitreal collagen fibers, are involved. Recent evidence suggests that hyalocytes, the vitreous resident myeloid cells [11, 12], may be essential participants in the course of proliferative vitreoretinal disease. Hyalocytes are distributed largely within the vitreous cortex abutting the retinal surface [13], in immediate proximity to RNV sites. We have previously demonstrated in a RNV mouse model that myeloid cells cluster in the vicinity of RNV [14] and may influence their formation [15]. Moreover, transcriptional and single-cell protein analysis of human RNV have revealed an abundance of antigen-presenting cells, most likely hyalocytes, which bear the potential of myofibroblastic transdifferentiation [16]. Finally, advanced in vivo imaging techniques have identified macrophage-like cells (MLC) accumulating around RNV in the vitreoretinal interface (VRI) of DR patients [17]. While these observations are suggestive, the exact role of vitreous hyalocytes in the pathophysiology of PDR remains largely unknown.

In this study, we report a high-dimensional molecular characterization of hyalocytes from the diabetic vitreous compared to controls performed with the aim to better assess their potential role in PDR pathophysiology. Our transcriptional analysis reveals enhanced inflammatory responses by hyalocytes, which appear to contribute, as a so far underestimated factor, to the cytokine pool within the PDR vitreous. The data further indicate an expression shift in hyalocytes in PDR towards a proangiogenic phenotype, suggesting they may mediate RNV formation. This could explain the misdirected growth of RNV towards the preretinal vitreous and alleviated RNV formation following vitrectomy. Finally, our data suggest a role of vitreous macrophages in erythrophagocytosis and removal of vitreous hemorrhage (VH) debris. The results of our study may pave the way for novel immunomodulatory approaches in the treatment of end-stage diabetic vitreoretinal disease.

## Materials and methods

### Patients’ characteristics

A total of 55 patients were included in this study. Only patients with no history of previous vitreoretinal surgery, concurrent vitreoretinal disease, or anti-VEGF therapy within the last three months were enrolled. Three control patients had type II diabetes without evidence of DR.

Adaptive optics scanning light ophthalmoscopy (AOSLO) imaging was performed on two patients (a 32-year-old healthy control [18] and a 26-year-old PDR patient [17]), as described previously [18]. The other 53 patients underwent vitrectomy for complications of PDR, macular pucker (MP) or macular hole (MH) between 2018 and 2021 (demographics and clinical characteristics summarized in Tables 1 and 2). RNA-Sequencing (RNA-Seq) analysis was performed on 21 samples from 30 patients (Tbl. 1). In order to exclude possible confounding effects of Red Blood Cell (RBC) lysis buffer treatment on the expression profile of vitreous hyalocytes, four pooled samples were processed for a preliminary analysis. For each final sample, the vitreous specimens of three to four MP or MH patients were pooled, divided in two equal halves and either subject to lysis (“+ lysis”, similar to diabetic samples in the main analysis) or analyzed without lysis treatment (“- lysis”, Tbl. 1). Eight samples from eight patients with PDR (mean age 57 ± 15.4 years) were compared to nine control samples from nine patients (MP and MH, mean age 75 ± 6.2 years) in the main analysis.

**Table 1:** Characteristics of patient samples included in RNA-Sequencing analysis

**Preliminary analysis**													
Sample #	Age	Sex	DM Type	Ocular Diagnosis	VH	TRD	Relevant systemic disorders	Lens status	PVD	Previous anti-VEGF treatment	Previous PRP treatment	Cell count	RNA concentration (pg/µl)
												+/- lysis	+/- lysis
1	83	F	NA	MP	NA	NA	asthma	phakic	no	NA	NA	90/264	71/116
	68	M	II	MP	NA	NA	-	phakic	no	NA	NA		
	69	F	NA	MP	NA	NA	-	phakic	no	NA	NA		
2	79	F	NA	MP	NA	NA	-	phakic	yes	NA	NA	94/86	104/154
	68	F	NA	MP	NA	NA	-	phakic	no	NA	NA		
	76	F	NA	MP	NA	NA	asthma, st. p. fibromyalgia, st. p. borreliosis	phakic	no	NA	NA		
	78	F	NA	MP	NA	NA	-	phakic	no	NA	NA		
3	61	F	NA	MH	NA	NA	-	phakic	no	NA	NA	79/139	84/11
	74	F	NA	MP	NA	NA	st. p. chemotherapy for breast carcinoma, Parkinson’s	phakic	no	NA	NA		
	82	M	NA	MP	NA	NA	-	phakic	no	NA	NA		
4	81	F	NA	MP	NA	NA	rheumatoid arthritis	pseudophakic	yes	NA	NA	139/147	43/85
	67	F	NA	MP	NA	NA	-	phakic	no	NA	NA		
	76	F	NA	MH	NA	NA	-	phakic	no	NA	NA		
**Main analysis**													
Sample #	Age	Sex	DM Type	Ocular Diagnosis	VH	TRD	Relevant systemic disorders	Lens status	PVD	Previous anti-VEGF treatment	Previous PRP treatment	Cell count	RNA concentration (pg/µl)
5	77	M	II	PDR	+	+	-	phakic	no	no	yes	280	38
6	40	M	I	PDR	+	+	-	phakic	no	no	yes	2300	322
7	56	M	II	PDR	+	+	-	phakic	no	no	no	980	49
8	29	F	I	PDR	+	+	-	phakic	no	no	no	1800	82
9	66	F	II	PDR	+	-	-	pseudophakic	yes	> 3 months	yes	454	68
10	62	M	I	PDR	-	+	st. p. kidney and pancreas transplantation	pseudophakic	no	no	yes	723	64
11	63	M	II	PDR	+	+	renal insufficiency	phakic	no	no	no	1077	211
12	64	F	II	PDR	+	-	-	pseudophakic	no	no	yes	512	86
13*	79	F	II	MP	NA	NA	asthma	pseudophakic	no	NA	NA	944	109
14	72	M	NA	MP	NA	NA	-	pseudophakic	yes	NA	NA	291	142
15	73	M	NA	MP	NA	NA	-	pseudophakic	no	NA	NA	464	161
16	73	F	NA	MP	NA	NA	fibromyalgia	pseudophakic	no	NA	NA	701	73
17*	68	M	NA	MH	NA	NA	psoriasis	phakic	no	NA	NA	424	73
18	88	M	NA	MH	NA	NA	-	pseudophakic	no	NA	NA	315	301
19	78	F	NA	MH	NA	NA	st. p. giant cell arteritis, celiac disease	phakic	yes	NA	NA	550	110
20	69	M	NA	MH	NA	NA	-	phakic	no	NA	NA	164	174
21	72	F	NA	MH	NA	NA	-	pseudophakic	no	NA	NA	240	78

DM, diabetes mellitus. VH, vitreous hemorrhage. TRD, tractional retinal detachment. PVD, posterior vitreous detachment. VEGF, vascular endothelial growth factor. PRP, panretinal photocoagulation. RNA, ribonucleic acid. NA, not applicable. MP, macular pucker. MH, macular hole. PDR, proliferative diabetic retinopathy. Two control samples (one MP and one MH, marked by an asterisk here) analyzed in a previous study [11] were processed anew and their newly generated transcriptional profiles compared to the data of the old sequencing batch. As no batch effect was present between the samples from the first analysis and the newly available sequencing profiles (data not shown), transcriptional data of the published control group of nine samples was processed for this study, too

**Table 2:** Characteristics of patient samples included in protein analysis

**ELISA**											
Sample #	Age	Sex	DM Type	Ocular Diagnosis	VH	TRD	Relevant systemic disorders	Lens status	PVD	Previous anti-VEGF treatment	Previous PRP treatment
1	28	M	I	PDR	+	+	-	phakic	no	no	yes
2	20	F	I	PDR	+	+	-	phakic	yes	no	yes
3	61	M	I	PDR	+	+	renal insufficiency	pseudophakic	no	no	yes
4	45	F	II	PDR	+	+	renal insufficiency	phakic	no	> 3 months	yes
5	45	F	II	PDR	+	+	-	phakic	no	no	no
6	67	F	II	PDR	+	+	-	phakic	no	no	yes
7	70	F	NA	MH	NA	NA	-	phakic	no	NA	NA
8	66	M	NA	MH	NA	NA	-	phakic	no	NA	NA
9	72	M	NA	MH	NA	NA	-	phakic	no	NA	NA
10	49	F	NA	MH	NA	NA	-	phakic	no	NA	NA
11	69	F	NA	MH	NA	NA	st. p. thyrodectomy for thyroid cancer	phakic	no	NA	NA
12	69	M	NA	MH	NA	NA	st. p. cryprogenic pneumonia	phakic	no	NA	NA
**Multiplex protein analysis**											
Sample #	Age	Sex	DM Type	Ocular Diagnosis	VH	TRD	Relevant systemic disorders	Lens status	PVD	Previous anti-VEGF treatment	Previous PRP treatment
13	35	M	I	PDR	+	+	renal insufficiency	phakic	no	no	yes
14	64	M	II	PDR	-	-	-	phakic	no	> 3 months	yes
15	52	F	II	PDR	+	+	-	phakic	no	no	yes
16	41	M	I	PDR	+	+	-	phakic	no	no	yes
17	20	F	I	PDR	+	+	-	phakic	yes	no	yes
18	33	M	I	PDR	+	+	-	phakic	no	> 3 months	yes
19	61	M	I	PDR	+	+	renal insufficiency	pseudophakic	no	no	yes
20	58	F	II	PDR	+	+	-	phakic	no	no	yes
21	45	F	II	PDR	+	+	renal insufficiency	phakic	no	> 3 months	yes
22	24	F	NA	MH	NA	NA	-	phakic	no	NA	NA
23	62	F	NA	MH	NA	NA	-	phakic	no	NA	NA
24	69	F	NA	MH	NA	NA	-	phakic	no	NA	NA
25	67	F	NA	MH	NA	NA	Graves’ disease	phakic	no	NA	NA
26	66	M	NA	MH	NA	NA	-	phakic	no	NA	NA
27	72	M	NA	MH	NA	NA	-	phakic	no	NA	NA
28	49	F	NA	MH	NA	NA	-	phakic	no	NA	NA
29	71	F	II	MH	NA	NA	possible giant cell arteriitis, renal insufficiency	pseudophakic	yes	NA	NA
30	69	F	NA	MH	NA	NA	st. p. thyrodectomy for thyroid cancer	phakic	no	NA	NA
31	69	M	NA	MH	NA	NA	st. p. cryprogenic pneumonia	phakic	no	NA	NA

DM, diabetes mellitus. VH, vitreous hemorrhage. TRD, tractional retinal detachment. PVD, posterior vitreous detachment. VEGF, vascular endothelial growth factor. PRP, panretinal photocoagulation. PDR, proliferative diabetic retinopathy. NA, not applicable. MH, macular hole

For protein analysis by enzyme-linked immunosorbent assay (ELISA) or multiplex immunoassay, 31 undiluted vitreous samples were obtained at the start of vitrectomy (before intraocular fluid infusion) from the mid-vitreous of 23 patients (samples from eight patients were processed for both readouts) and centrifuged at 500 x *g* for 20 min at 4 °C. Corresponding plasma specimens were collected before or during surgery by peripheral venous puncture, centrifuged (3000 x *g* for 15 min at 4 °C) and, like vitreous samples, stored at -80 °C until processing.

Ethics approval was granted by the local Ethics Committee and a written informed consent was obtained from each patient prior to surgery. All research adhered to the tenets of the Declaration of Helsinki.

### Fluorescence-activated cell sorting (FACS)

Vitreous tissue samples were collected in vitrectomy bags during surgery and processed for cell isolation within two hours of resection, according to a previously published protocol [11] adapted for the specificities of diabetic vitreous. For half of the samples in the preliminary analysis (see Table 1) and for diabetic samples, 1 ml of RBC lysis buffer (Thermo Fisher Scientific) was added for erythrocyte lysis. Samples were stained for cluster of differentiation (CD) 45 (BV421, anti-human, 1:100, BioLegend), CD11b (FITC, anti-human, 1:100, BioLegend), CX_3_C motif chemokine receptor 1 (CX_3_CR1, PE-Cy7, anti-human, 1:200, BioLegend), and with the anti-human Mature Macrophages (MatMac) antibody, an ED2-like (ectodermal dysplasia 2) marker for resident macrophages (eFluor660, anti-human, 1:100, eBioscience). Finally, cells were processed for sorting on the MoFlo Astrios EQ Cytometer (Beckman Coulter). Hyalocytes were isolated as CD45^+^CD11b^+^CX3CR1^+^MatMac^+^ cells.

### Total RNA extraction and RNA-Seq library preparation

RNA extraction, library preparation and RNA-Seq were conducted at the Genomics Core Facility “KFB - Center of Excellence for Fluorescent Bioanalytics” (University of Regensburg, Germany), according to previously published protocols [11, 16].

### Bioinformatics

Sequencing data were analyzed on the Galaxy web platform (usegalaxy.eu [19]), as previously described by our group [20]. Transcripts with log2 fold change (log2FC) > 2 or < -2 and adjusted *p*-value < 0.05 were considered differentially expressed genes (DEG). Gene ontology (GO) analysis for clusters related to biological processes (BP) was performed based on all DEG in hyalocytes from PDR patients vs. controls.

### Multiplex immunoassay

Cytokine levels were measured in vitreous and corresponding plasma samples from nine PDR (mean age 45 ± 14.7 years) and 10 MH (control) patients (mean age 62 ± 14.8 years, Tbl. 2). A multiplex electrochemiluminescence assay (V-Plex Human Biomarker 54-Plex Kit, Meso Scale Discovery) was used according to manufacturer’s instructions. By combination of electrochemiluminescence and multi-array technologies, the levels of 54 cytokines were simultaneously measured including interleukin (IL)-6, IL-8, IL-15, monocyte chemoattractant protein 1 (MCP-1), placental growth factor (PlGF), VEGF-A. This panel was selected to assess the expression of factors previously identified as relevant in the progression of PDR or DEG in hyalocytes from PDR patients based on the RNA-Seq results of this study. For statistical analysis, all values below the detection limit were assigned to half the respective values of the detection limit.

### Enzyme-linked immunosorbent assay (ELISA)

Vitreous and plasma levels of ANGPT2 were measured in samples from six PDR (mean age 44 ± 18.2 years) and six MH (control) patients (mean age 66 ± 8.5 years, Tbl. 2) with the Human Angiopoietin-2 Quantikine ELISA (R&D Systems) according to manufacturer’s instructions.

### Cultivation and staining of porcine hyalocytes

Porcine eyes were obtained from a local abattoir. In terms of development, anatomy and morphology, these are considered representative for the human situation [21]. Vitreous was extracted from six eyes and placed in Dulbecco’s Modified Eagle Medium (DMEM, Gibco). On the next day, peripheral blood was obtained from healthy human donors by venous puncture and centrifuged at 3000 rpm for 10 min at 4 °C to obtain erythrocytes. One drop of erythrocyte concentrate was added to each vitreous sample. On day 3, specimens were fixed in methanol for 10 min at -20 °C prior to blocking for one hour at room temperature (RT) in a solution of 1% bovine serum albumin (Roth) and 5% normal donkey serum (Biozol) in phosphate-buffered saline Triton-X 0.1% (Gibco). For immunohistochemistry (IHC), cells were incubated with ionized calcium-binding adapter molecule 1 (IBA-1, abcam) and CD235a (Thermo Fischer Scientific) primary antibodies for one hour at RT. Primary antibodies were omitted for negative control. Incubation with secondary antibodies (Alexa 647, Life Technologies; Alexa 488, Invitrogen) and Phalloidin 5(6)-Tetramethylrhodaminisothiocyanate (TRITC, Sigma-Aldrich) was performed for one hour at RT. Nuclei were counterstained with 4′,6-Diamidin-2-phenylindol (DAPI) prior to embedding in Fluorescence Mounting Medium (Agilent Dako). Representative images were taken on a Leica TCS SP8 Confocal System coupled to a Leica DMi8 inverted microscope.

### Statistical analysis

Statistical analysis was performed using GraphPad Prism (GraphPad Software, Version 10.2.3). For multiplex immunoassay analysis, one-way ANOVA and subsequent multiple comparison testing was performed. For ANGPT2 expression analysis by ELISA, a Mann-Whitney test was applied. The following significance levels were considered: *p < 0.05, **p < 0.01, ***p < 0.001, ****p < 0.0001).

## Results

### Analysis workflow

**Fig. 1:**
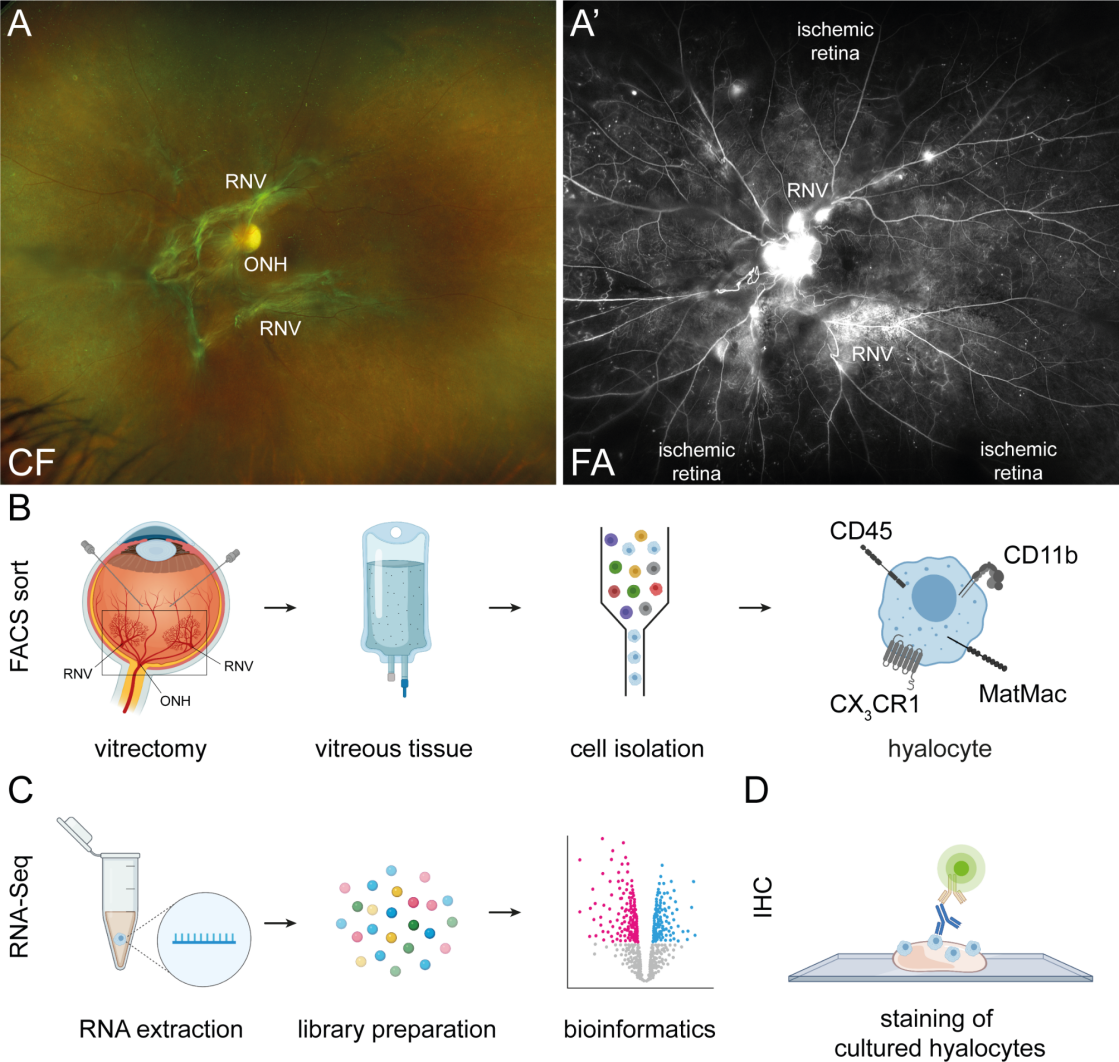
Experimental workflow. Diagnosis was based on a thorough funduscopic exam documented by fundus photography (CF, color fundus, **A**.), spectral domain optical coherence tomography (OCT) and fluorescence angiography (FA, **A’**.), for proliferative diabetic retinopathy (PDR) patients (black areas are not sufficiently supplied with blood and therefore ischemic). **B**. Vitreous samples were obtained by vitrectomy from PDR and control patients. The black rectangle is to illustrate the part of the bulb imaged by CF in **A**. and FA in **A’**. Hyalocytes were isolated from vitreous tissue by flow cytometry (fluorescence-activated cell sorting, FACS) as CD45^+^CD11b^+^CX3CR1^+^MatMac^+^ cells and were further processed for **C**. RNA extraction and library preparation. RNA-Sequencing (RNA-Seq) data were analyzed bioinformatically. **D**. For visualization of hyalocytes, porcine vitreous tissue was cultured and stained by immunohistochemistry (IHC). ONH. Optic nerve head. RNV, retinal neovascularization

**Fig. 2:**
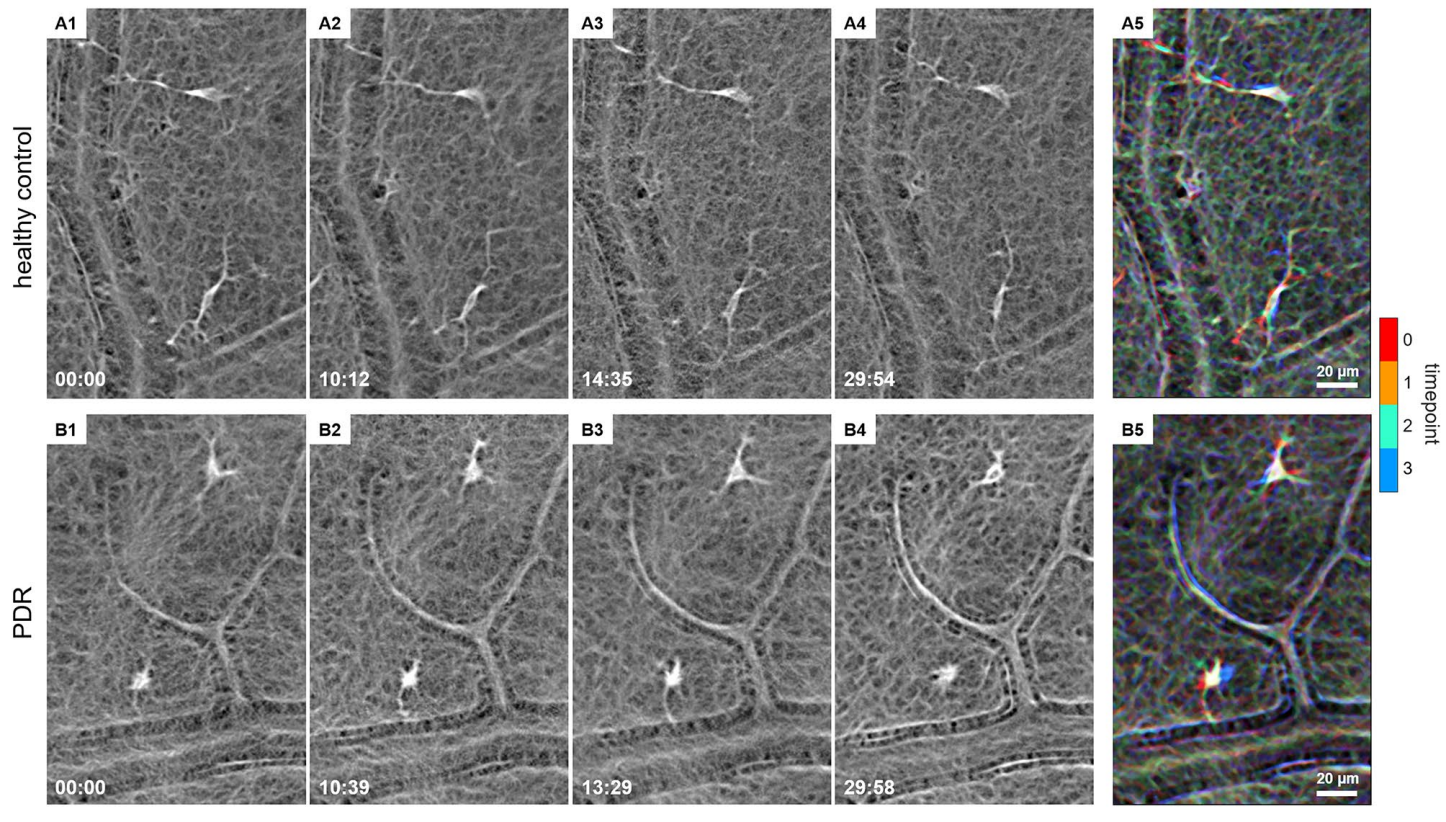
Adaptive optics scanning light ophthalmoscopy (AOSLO) imaging of macrophage-like cells in the vitreoretinal interface. Two adjacent hyalocytes with different movement behaviors over 30 min of AOSLO imaging in (top row, **A1-A4**; modified from [18]) a 32-year-old healthy control and (bottom row, **B1-B4**) a 26-year-old proliferative diabetic retinopathy (PDR) patient. **A5 & B5.** Chromo-temporal map composites of the four time points over 30 min, which indicate movement of cell bodies and processes across the imaging period. Time of acquisition in the lower-left corner is displayed in mins:secs. A video of the entire imaging region of interest, which includes these four cells, is shown in Additional File 1

Diagnosis for the 55 included patients was based on a thorough funduscopic exam (Fig. 1A), spectral domain optical coherence tomography (OCT) and fluorescence angiography (FA, HRA2, Heidelberg Engineering) for PDR patients (Fig. 1A’). Samples from 30 patients undergoing vitrectomy for PDR, MP or MH were processed for RNA-Seq analysis (Tbl. 1). Hyalocytes were isolated from vitreous tissue by flow cytometry (Figs. 1B, [11]) specifically targeting resident immune cells by utilizing, among others, the MatMac marker for tissue-specific macrophages. This antibody was applied to exclude any potential contamination with blood-derived monocytes owing to possible surgically induced microbleeds, as infiltrating leukocytes generally lack the antigen [22, 23]. In the following, sorted hyalocytes were analyzed by RNA-Seq (Fig. 1C). Staining by IHC was performed on hyalocytes (Fig. 1D) isolated from porcine vitreous tissue. MLC in the VRI of a healthy control and a PDR patient imaged by AOSLO are shown in Fig. 2 and Additional File 1.

### Effect of RBC lysis on the transcriptional profile of human hyalocytes

In our study, RBC lysis proved indispensable for isolation of hyalocytes from the blood-tinged diabetic vitreous, but unnecessary to process control vitreous. Furthermore, a lysis-induced reduction of cell count in single non-diabetic samples under the limit of feasible transcriptional analysis was observed in preparation for this study.

Although lysed samples in our preliminary analysis showed a trend to a lower cell count (mean count: 100.5 ± 26.4 cells vs. 159.0 ± 75.1 cells, *p* = 0.22) and lower RNA concentration (mean concentration: 75.5 ± 25.6 pg/µl vs. 91.5 ± 60.6 pg/µl, *p* = 0.65), no distinct effect of lysis on the RNA profile of hyalocytes could be inferred, as highlighted by the conducted principal component analysis (PCA, Additional File 2A) and heatmap visualization (Additional File 2B). Based on the negligible effect of RBC lysis treatment on hyalocyte transcriptional data, further analysis was performed comparing lysed diabetic samples and non-lysed control samples (MP and MH). MP and MH hyalocytes are considered comparable in their transcriptional signature [11], which prompted us to process them together.

### Transcriptional profiling of human diabetic hyalocytes

Diabetic samples tended to yield a higher amount of hyalocytes in comparison to control samples (1015.8 ± 703.6 vs. 454.8 ± 246.8 cells, *p* = 0.063), while mean concentration of RNA extracted from isolated hyalocytes was comparable (115.0 ± 99.3 vs. 135.7 ± 72.5 pg/µl, *p* = 0.636). A mean total number of 43.7 million (± 4.3) raw reads per sample was obtained from diabetic hyalocytes, while 37.8 million (± 6.5) raw reads per sample were detected for control hyalocytes (*p* = 0.045).

**Fig. 3:**
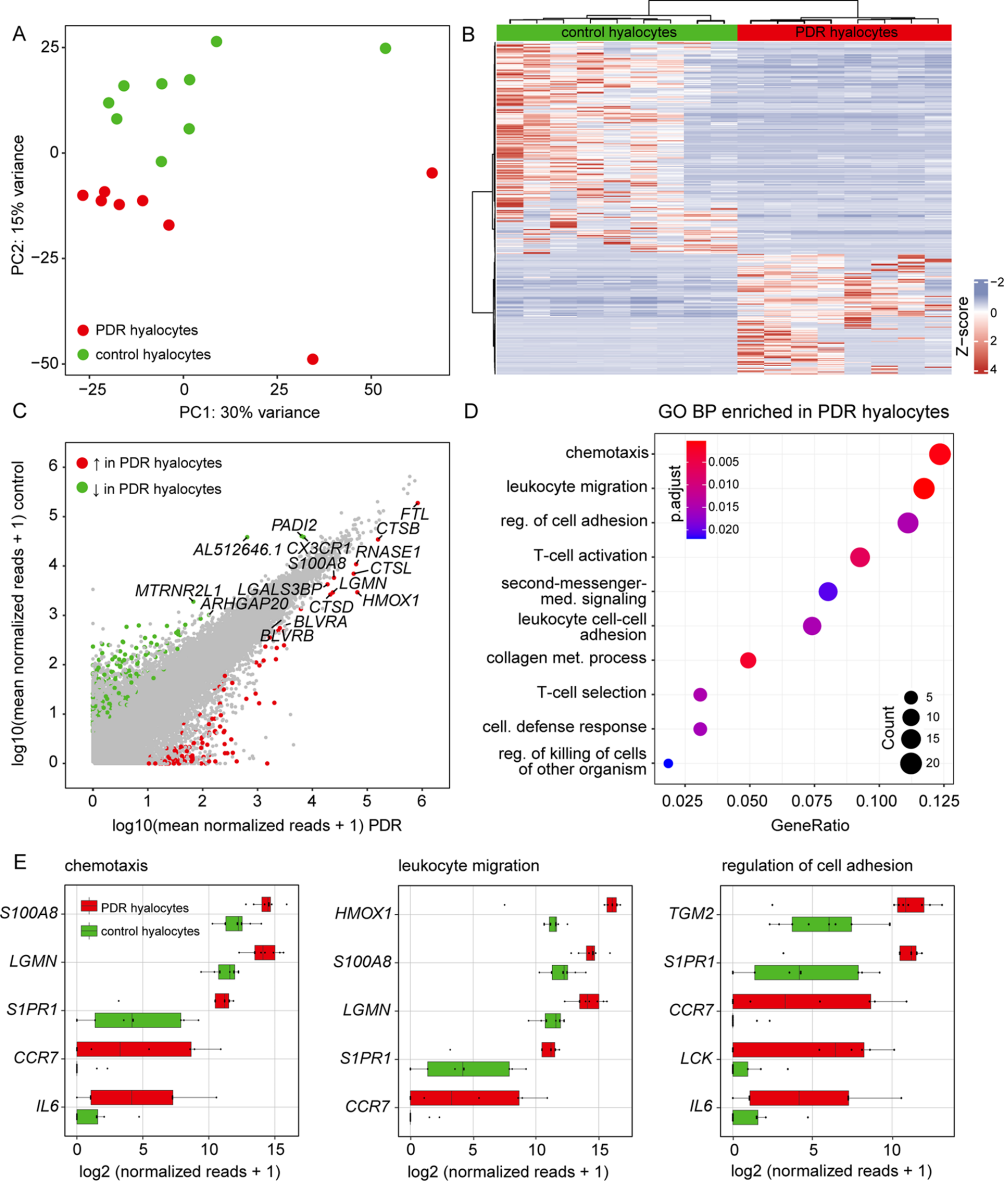
Transcriptional signature of diabetic hyalocytes. (**A**) A Principal Component Analysis (PCA) plot demonstrates the distribution of hyalocyte samples isolated from the vitreous of patients with PDR (red dots) and control samples (green dots). **(B)** Heatmap of differentially expressed genes (DEG, log2FC > 2, adjusted *p* value < 0.5, sorted according to the logFC) between hyalocytes isolated from the vitreous of PDR patients and control hyalocytes. Color coding of the transcripts according to the z-score (deviation from a gene’s mean expression in standard deviation units). **(C)** Readplot of DEG in diabetic hyalocytes (upregulated genes in red, downregulated genes in green, not differentially expressed genes in grey; the most strongly expressed genes from each group are labeled; also labeled are the biliverdin reductases A and B, *BLVRA* and *BLVRB*). **(D)** Top 10 most significantly upregulated Gene Ontology (GO) biological process (BP) clusters on the basis of all DEG in diabetic hyalocytes. Color coding of the dots according to the adjusted *p* value, size of the dots according to the count of transcripts associated with the respective GO term. **(E)** Top five most highly expressed transcripts in the three most enriched GO terms from D

A total of 43,278 transcripts with at least one read in at least one sample were obtained. PCA revealed clear separation of diabetic and control hyalocytes’ transcriptomes on the first two principle components indicating substantial differences between the two groups (Fig. 3A). Despite within-group heterogenity visible in the generated DEG heatmap, a distinct pattern of transcriptome differences was evident between both entities (Fig. 3B). Comparative analysis of the transcriptome of diabetic and control hyalocytes revealed 126 differentially upregulated and 222 differentially downregulated genes in diabetic samples relative to controls (Fig. 3C). Notably, among the most strongly upregulated genes in diabetic hyalocytes ranked a number of factors implicated in inflammatory (cathepsin (CTS) B (*CTSB*), *CTSL*, *CTSD* and S100 calcium-binding protein A8 (*S100A8*)), but also anti-inflammatory responses (ferritin light chain, *FTL*), or both (legumain (*LGMN*)).

To gain more insight into BP diabetic hyalocytes are involved in, we performed a GO cluster analysis of all DEG (Fig. 3D). According to our data, DEG mostly contributed to processes like “chemotaxis” (GO:0006935) and “leukocyte migration” (GO:0050900), but also “regulation of cell adhesion” (GO:0030155) and “collagen metabolic process” (GO:0032963). *S100A8* was the most highly expressed factor in the cluster “chemotaxis” and the second-most highly expressed factor in “leukocyte migration” (Fig. 3E).

**Fig. 4:**
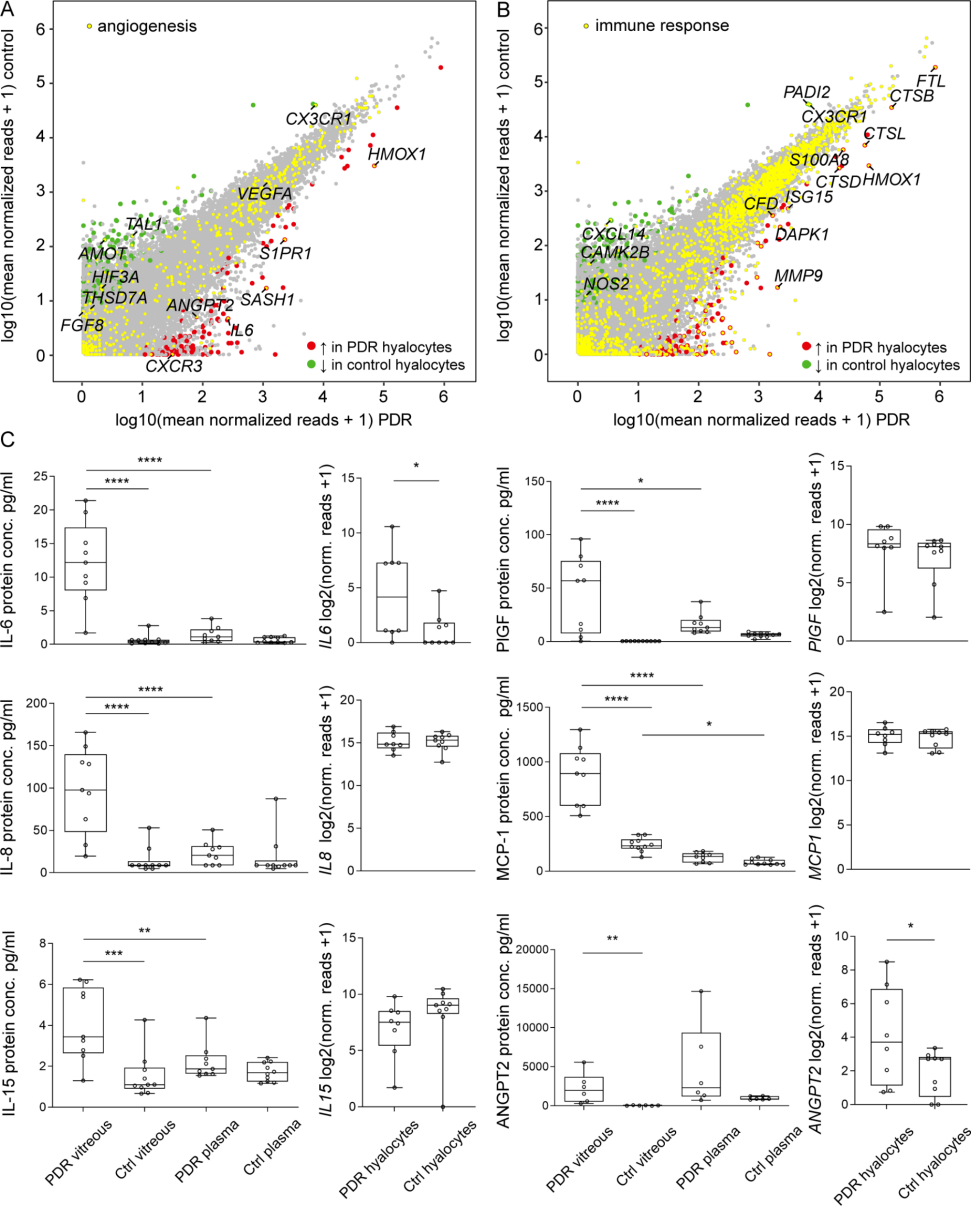
“Angiogenesis” and “immune response”-associated genes expressed in hyalocytes from proliferative diabetic retinopathy (PDR) patients. **(A)** Readplot of differentially expressed genes (DEG) in diabetic hyalocytes (upregulated genes in red, downregulated genes in green, not differentially expressed genes in grey; factors associated with the Gene Ontology (GO) biological process (BP) “angiogenesis” are highlighted in yellow; the most strongly upregulated “angiogenesis”-related genes are labeled). **(B)** Readplot of DEG in diabetic hyalocytes (upregulated genes in red, downregulated genes in green, not differentially expressed genes in grey; factors associated with the GO BP “immune response” are highlighted in yellow; the most strongly upregulated “immune response”-related genes are labeled). **(C)** Left-hand side panels show boxplots for multiplex immunoassay analysis of interleukin (IL)-6, placental growth factor (PlGF), IL-8, monocyte chemoattractant protein-1 (MCP-1) and IL-15 protein expression or enzyme-linked immunosorbent analysis (ELISA) of angiopoietin-2 (ANGPT2) protein expression in PDR vitreous and plasma and control (Ctrl) vitreous and plasma samples. *p < 0.05, **p < 0.01, ***p < 0.001, ****p < 0.0001 (one-way ANOVA and consequent analysis of multiple comparisons conducted for multiplex immunoassay analysis and Mann-Whitney-Test performed for ELISA analysis). Corresponding gene expression analysis for *IL6*, *PlGF*, *IL8*, *MCP1* (= *CCL2*), *IL8* (= *CXCL8*) and *ANGPT2* in PDR and control hyalocytes is shown on the right, respectively

Since angiogenesis and immune response are key processes in the pathogenesis of PDR [24], we took a closer look at genes involved in both processes. We found heme oxygenase 1 (*HMOX1*) involved in hemoglobin catabolism, but also *IL6* and angiopoietin-2 (*ANGPT2*) to be among the most strongly expressed factors in “angiogenesis” in hyalocytes from PDR patients (Fig. 4A). For “immune response”, besides the already established factors *FTL*, *CTSB*, *CTSL*, *CTSD* and *S1008*, matrix metalloproteinase 9 (*MMP9*) was among the PDR-associated factors in hyalocytes that stood out (Fig. 4B).

### Protein analysis

According to the multiplex immunoassay, IL-6, which was overexpressed in PDR hyalocytes on the RNA level (log2FC = 4.24, adjusted *p* = 0.01), was also strongly enriched in PDR vitreous in comparison to control vitreous and when compared to corresponding plasma on the protein level (Fig. 4C). PlGF was also significantly upregulated in PDR vitreous in comparison to both control vitreous and corresponding plasma. However, these changes did not correspond to a significant enrichment of the respective gene in diabetic hyalocytes (log2FC = 0.83, adjusted *p* = 0.74, Fig. 4C). The same was the case for IL-8 (log2FC = 0.17, adjusted *p* = 0.97), MCP-1 (log2FC = 0.25, adjusted *p* = 0.95) and IL-15 (log2FC = -1.00, adjusted *p* = 0.72, Fig. 4C). VEGF was detected on the protein level by two assays, which both demonstrated an upregulation in the diabetic vitreous when compared to control vitreous (**p < 0.01). In one of the assays, an upregulation against corresponding plasma of PDR patients was detected (**p < 0.01). Of note, gene expression of *VEGFA* did not differ significantly between hyalocytes isolated from the PDR and control vitreous, indicating that other ocular cell types are likely the source of increased VEGF protein levels in the diabetic vitreous.

As assessed by ELISA, ANGPT2, a DEG in PDR hyalocytes when compared to control hyalocytes (log2FC = 3.57, adjusted *p* = 0.01), was significantly upregulated in the PDR vitreous in comparison to control vitreous, too (Mann-Whitney test, Fig. 4C).

### Erythrophagocytosis in hyalocytes

**Fig. 5:**
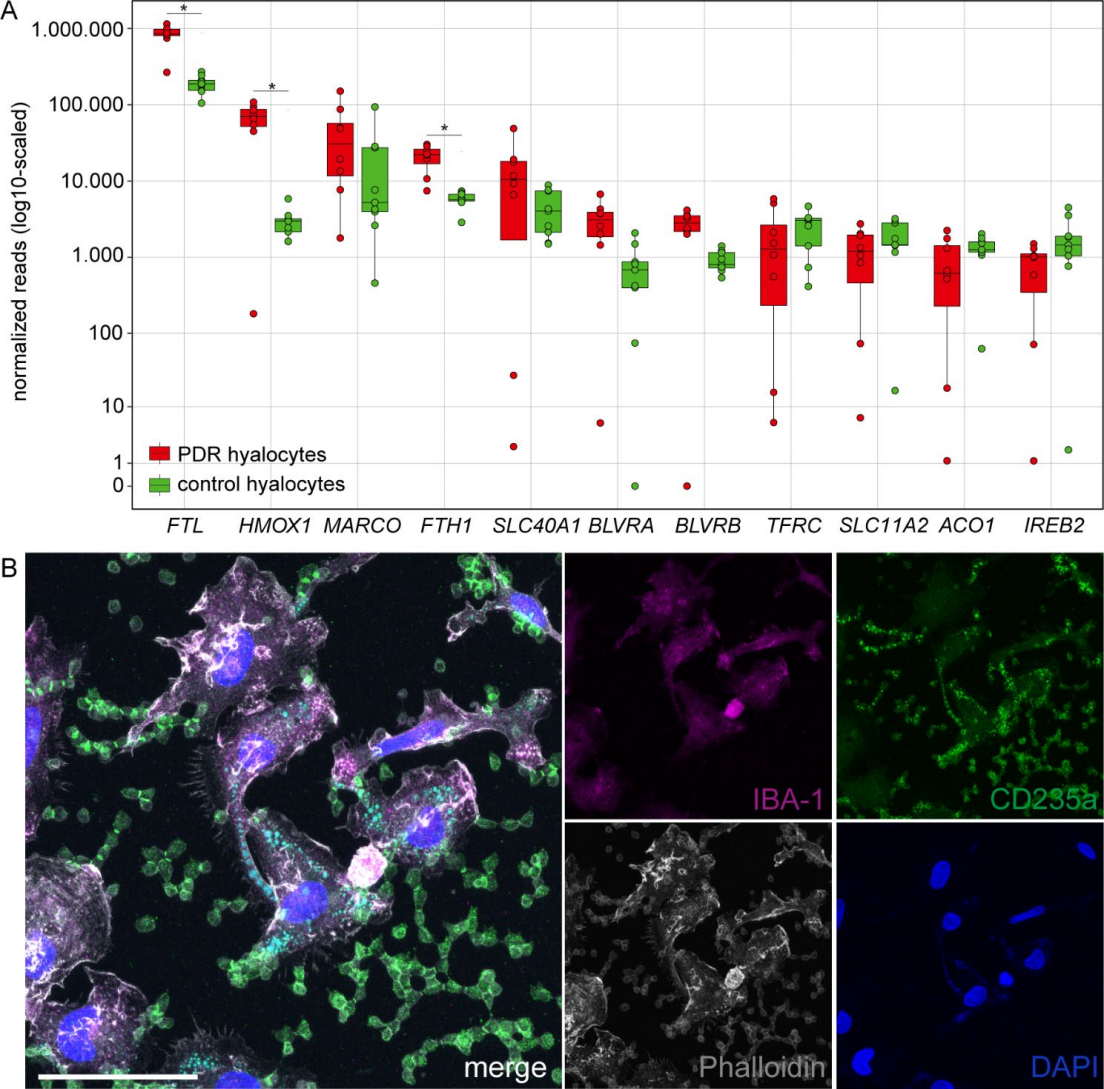
Erythrophagocytosis in hyalocytes. **(A)** Boxplots of the expression of marker genes of erythrophagocytosis and iron regulator genes in proliferative diabetic retinopathy (PDR, red) and control (green) hyalocytes. *p < 0.05. **(B)** Immunohistochemical staining of cultured porcine hyalocytes for ionized calcium-binding adaptor molecule 1 (IBA-1), cluster of differentiation 235a (CD235a) and Phalloidin. Nuclei are counterstained with 4′,6-Diamidin-2-phenylindol (DAPI). Within the cytoplasm of hyalocytes, vesicles containing CD235a- (= erythrocyte debris), but also DAPI-stained particles (= digested nuclei) were observed. Scale bar corresponds to 50 μm

According to our transcriptional analysis, *HMOX1*, an important factor in heme catabolism [25, 26], was one of the most highly expressed genes in diabetic hyalocytes when compared to control hyalocytes. This prompted us to elucidate further marker genes of erythrophagocytosis and iron regulator genes [25, 27] in the data set. Beside *FTL* and *HMOX1*, ferritin heavy chain 1 (*FTH1*) was also significantly upregulated in diabetic hyalocytes. Other factors, such as the biliverdin reductases A and B (*BLVRA* and *BLVRB*), showed a trend (*BLVRA*: log2FC = 1.78, adjusted *p* = 0.30, *BLVRB*: log2FC = 1.42, adjusted *p* = 0.32) towards an enrichment in PDR hyalocytes (Figs. 2C and 5A).

In order to assess the potential of hyalocytes to phagocytose erythrocytes, as suggested by our sequencing results, we next examined cultured porcine hyalocytes. Hyalocytes seemed to engulf erythrocytes, which could clearly be distinguished within the Phalloidin-stained cytoskeleton of the immune cells. Within the cytoplasm of IBA1-positive hyalocytes, we observed vesicles containing CD235a-stained, most probably erythrocyte debris, but also DAPI-positive particles to be considered digested nuclei (Fig. 5B). Negative controls are shown in Additional File 3 (for IBA-1: **A.**, for CD235a: **B.**). These data suggest that hyalocytes have the potential to remove damaged erythrocytes, which may be of critical importance for diabetic VH clearance.

## Discussion

More than one third of PDR cases deteriorate despite complete panretinal photocoagulation or continuous anti-VEGF treatment [28]. Thus, investigation of the molecular and cellular mechanisms underlying end-stage DR pathophysiology is indispensable to prevent severe vision loss. In this study, we performed an in-depth transcriptional characterization of hyalocytes isolated from the vitreous of PDR patients to assess the role of this specialized immune cell population in disease progression. We demonstrate that hyalocytes contribute to a proinflammatory and proangiogenic milieu in the diabetic vitreous by expressing several cytokines but may also play a role in VH clearance. In addition to a presumed role in tractional retinal detachment (TRD) by hyalocyte-to-myofibroblast transdifferentiation, as suggested by us previously [16], our current data implicate the importance of hyalocytes in angiogenesis and red blood cell cleanup, two further hallmarks of vision-threatening PDR.

The transcriptional analysis of 43,278 genes revealed a distinct expression signature of diabetic hyalocytes when compared to control hyalocytes. GO cluster analysis demonstrated that DEG in diabetic hyalocytes mainly contribute to processes like “chemotaxis” and “leukocyte migration” highlighting the importance of innate immune responses in the course of severe vitreoretinal disease. The more active immunological state of PDR hyalocytes was supported by AOSLO imaging of hyalocytes, which in PDR appeared amoeboid-shaped in comparison to the slender, ramified and quiescent MLC in a healthy patient. Besides, DEG were enriched in processes like “regulation of cell adhesion” and “collagen metabolic process”, which suggests an involvement of diabetic hyalocytes in wound repair and would be in line with previous findings on hyalocyte-to-myofibroblast transdifferentiation in PDR [16].

Next, we examined closely two key pathways in PDR pathophysiology – angiogenesis and immune response. In contrast to *VEGFA*, *IL6* and *ANGPT2* were among the most strongly enriched factors for the term “angiogenesis” in hyalocytes from PDR patients suggesting these cells as an origin for proinflammatory and proangiogenic cytokines in the diabetic vitreous. IL-6 has been previously shown to be enriched in the diabetic vitreous [29], which may correspond to disease severity [30]. In our study, IL-6 was strongly overexpressed in PDR vitreous samples in comparison to control vitreous and corresponding plasma, which implies a local production by vitreal and/or retina cells. The potential of IL-6 signaling inhibition is currently being explored in experimental and clinical trials for the treatment of DR-related complications [31]. *ANGPT2* also lined among the “angiogenesis”-related DEG in diabetic hyalocytes. The factor is known from routine clinical practice with the application of the faricimab antibody [32]. An overexpression of ANGPT2 in the diabetic vitreous on the protein level has been previously demonstrated [33] and was confirmed here. For other analytes, too, such as PlGF, IL-8, MCP-1, and IL-15, a strong upregulation in PDR vitreous samples when compared to control vitreous and corresponding plasma was detected, which is in line with previously published data [29, 34, 35]. However, these factors were not significantly enriched in diabetic hyalocytes implying alternative cell populations as the source for their abundance in the vitreous. It should further be critically considered that various cells of the retina known to express IL-6 (fibroblasts and pericytes) and ANGPT2 (endothelial cells, [36]) in the steady state more pronouncedly than hyalocytes may be the major contributors of both factors to the diabetic vitreous.

Among the 126 differentially upregulated genes in hyalocytes from PDR patients, several proinflammatory factors, such as *CTSB*, *CTSL*, *CTSD* and *S100A8*, stood out. Cathepsins have previously been implied in mediating an inflammatory response in macrophages, e.g. in carotid plaques [37], adipose tissue [38] and inflammatory bowel disease [39]. *S100A8*, together with *S100A9*, encodes for the S100A8/A9 protein complex calprotectin and its expression is upregulated in human choroidal neovascularization tissue [40]. Myeloid cells including macrophages express S100A8/A9 constitutively, while in inflammation, secretion of the protein is enhanced and known to stimulate cytokine release [41]. Inhibition of S100A8/A9 alleviates excessive cytokine production, which reveals its potential as a therapeutic target [42]. In addition, *FTL*, which has previously been determined as the second-most prominent transcript in human control hyalocytes [11], ranked among the top expressed factors in diabetic hyalocytes, also, with a much higher expression in the disease state. *FTL* encodes the light subunit of the ferritin protein, the main iron storage in the human body previously associated with anti-inflammatory responses in murine macrophages [43]. Since an iron overload and subsequent susceptibility to oxidative damage has been described for the PDR vitreous [44], it is tempting to speculate about a role of hyalocyte-derived *FTL* in neuroprotective iron reduction in the vitreous.

On another note, potential deleterious effects of activated hyalocytes in PDR conveyed by the expression of inflammatory and angiogenic factors such as IL-6 and ANGPT2 may well be in contrast to beneficial features of this cell population known, similarly to microglia, for its dual nature [45]. Along these lines, our transcriptional analysis revealed an upregulation of factors known from hemoglobin catabolism, such as *HMOX1*, *BLVRA* and *BLVRB*, in diabetic hyalocytes. The notion of hyalocytes’ phagocytic activity has been suggested as early as in the middle of the 20th century by Hamburg, who assumed that hyalocytes contribute to clearance of metabolic products [46]. By state-of-the-art imaging, hyalocytes have been shown here and previously [18] to continuously scan the environment with their protrusions in anticipation of harmful signals. Upon detection of danger, hyalocytes are likely to migrate and phagocytose the pathogen, akin to microglia of the central nervous system and in accordance with recent evidence that hyalocytes express factors important for phagocytosis, such as *MERTK* (MER proto-oncogene, tyrosine kinase), *CD74* and MHCII-associated genes (major histocompatibility complex class II, [11]). To assess hyalocytes’ potential to phagocytose erythrocytes, we exposed cultured porcine hyalocytes to erythrocytes and subsequently stained them for CD235a, an abundant protein in erythrocyte membranes. Our data demonstrate the capacity of hyalocytes to engulf and dispose of erythrocytes, a process known as erythrophagocytosis. Hereby, damaged/senescent erythrocytes are removed from circulation primarily by macrophages in the spleen, liver, and bone marrow [47]. For erythrocytes, CD47 is known as a “marker of self” preventing their premature clearance [48]. Further studies are needed, in order to elucidate if “eat me/don’t eat me” signals [49] to hyalocytes are also conveyed CD47-dependently and how hyalocytes decide whether they are going to embrace their advantageous functions with erythrophagocytosis, or involve in pernicious cytokine release in vitreoretinal disease.

Interpretation of our data is limited by several factors, for instance the use of samples from patients with vitreoretinal disease, namely MP or MH, as controls. However, MP and MH represent the most physiological fresh samples that can be obtained in clinical routine. Another important limiting factor in our study is the age difference between PDR and control patients. While MP and MH are regarded age-related disorders of the VRI, severe PDR commonly affects younger patients. Differences in age distribution in this study should therefore be critically considered. Further, it was not possible to obtain detailed information on the preoperative extent of avascularity and degree of neovascularization for PDR patients, since FA of sufficient quality was not feasible for the majority of patients due to view obscuration by VH. Lastly, the young age of pigs serving as a source for our in vitro model could also represent a confounder. However, since the vitreous of 8-10-month-old pigs as examined in our study, an age that in theory corresponds to human 18 years, has been reported to theoretically possess the viscoelastic properties of adult human vitreous [50], we consider this limitation negligible.

## Conclusions

In conclusion, transcriptional analysis of diabetic hyalocytes, protein analysis of PDR vitreous and immunohistochemical studies on cultured hyalocytes in our work reveal an enrichment of proinflammatory and proangiogenic factors, such as *IL6* and *ANGPT2*, in hyalocytes from PDR patients, which is conveyed in an abundance of both factors in the diabetic vitreous on the protein level. Our data further suggest an important role of hyalocytes in erythrophagocytosis, which may be critical in VH clearance in PDR, especially in non-vitrectomized eyes. As hyalocytes have previously been shown to transdifferentiate to myofibroblasts, this unique cell population of the vitreous may play a role in both critical complications of PDR: vitreous hemorrhage and tractional retinal detachment. Immunomodulation of hyalocytes may thus prove an essential novel therapeutic approach in diabetic vitreoretinal disease.

## Electronic supplementary material

Below is the link to the electronic supplementary material.

**Additional File 1 (Movie**,**.avi**). Adaptive optics scanning light ophthalmoscopy (AOSLO) time-lapse video of macrophage-like cells in the vitreoretinal interface. Two adjacent hyalocytes with different movement behaviors over 30 min of AOSLO imaging in (**A**) a 32-year-old healthy control and (**B**) a 26-year-old proliferative diabetic retinopathy (PDR) patient. Time of acquisition in the lower-left corner is displayed in mins:secs. Video is looped three times for better visualization

**Additional File 2 (Figure**,**.tif**). Impact of Red Blood Cell (RBC) Lysis on hyalocyte expression. In order to assess the effects of the RBC lysis procedure on the transcriptional profile of hyalocytes, we conducted a preliminary analysis of control samples (each pooled from the vitreous tissue of 3 to 4 patients, see Table 1), which were processed to equal parts for a treatment with and without lysis. **(A)** Principal Component Analysis (PCA) demonstrating distribution of the analyzed entities: samples processed with lysis (“+ lysis”, dark red dots) and non-lysed samples (“- lysis”, light blue dots). The only sample in the right part of the graph was designated as a relative outlier, as less mapped reads were assigned to this sample than to other analyzed samples. **(B)** Unsupervised heatmap of expressed genes sorted according to mean expression in all samples

**Additional File 3 (Figure**,**.tif**). Negative controls for immunohistochemistry. For negative controls, primary antibodies were omitted. Negative control for ionized calcium-binding adaptor molecule 1 (IBA-1, **A**) and cluster of differentiation 235a (CD235a) immunohistochemical staining (**B**) shown in Fig. 5B. Nuclei are counterstained with DAPI (4′,6-Diamidin-2-phenylindol). DAR647, donkey anti-rabbit Alexa Fluor 647. DAM488, donkey anti-mouse Alexa Fluor 488. Scale bars correspond to 100 μm

## Data Availability

Sequencing data generated in this study are available in the Gene Expression Omnibus (GEO) database under accession number GSE276892. For PDR samples processed for transcriptional analysis sample numbers in this dataset correspond to annotation in Table 1. For control samples analyzed elsewhere [11], GEO accession numbers are listed here (one number for each flow cell, three flow cells per sample): sample #14: GSM4437362, GSM4437363, GSM4437364; sample #15: GSM4437365, GSM4437366, GSM4437367; sample #16: GSM4437380, GSM4437381, GSM4437382; sample #18: GSM4437368, GSM4437369, GSM4437370; sample #19: GSM4437371, GSM4437372, GSM4437373; sample #20: GSM4437374, GSM4437375, GSM4437376; sample #21: GSM4437383, GSM44373834, GSM44373835.

## Abbreviations

AOSLO: Adaptive optics scanning light ophthalmoscopy
ANGPT2: Angiopoietin-2
BLVR: Biliverdin reductases
BP: Biological process
CD: Cluster of differentiation
CF: Color fundus
CTS: Cathepsin
CX_3_CR1: CX_3_C motif chemokine receptor 1
DAPI: 4′,6-Diamidin-2-phenylindol
DEG: Differentially expressed genes
DME: Diabetic macular edema
DMEM: Dulbecco’s Modified Eagle Medium
DR: Diabetic retinopathy
ECM: Extracellular matrix
ED2: Ectodermal dysplasia 2
ELISA: Enzyme-linked immunosorbent assay
FA: Fluorescence angiography
FACS: Fluorescence-activated cell sorting
FTH1: Ferritin heavy chain 1
FTL: Ferritin light chain
GO: Gene ontology
HMOX1: Heme oxygenase 1
IBA-1: Ionized calcium-binding adapter molecule 1
IHC: Immunohistochemistry
IL: Interleukin
LGMN: Legumain
log2FC: Log2 fold change
MatMac: Anti-human Mature Macrophages antibody
MCL: Macrophage-like cells
MCP-1: Monocyte chemoattractant protein 1
MERTK: MER proto-oncogene, tyrosine kinase
MH: Macular hole
MHCII: Major histocompatibility complex class II
MMP9: Metalloproteinase 9
MP: Macular pucker
OCT: Optical coherence tomography
ONH: Optic nerve head
PCA: Principal component analysis
PDR: Proliferative diabetic retinopathy
PlGF: Placental growth factor
PVD: Posterior vitreous detachment
S100A8: S100 calcium-binding protein A8
RBC lysis buffer: Red Blood Cell lysis buffer
RNA: Ribonucleic acid
RNA-Sequencing: RNA-Seq
rpm: Rounds per minute
RNV: Retinal neovascularization
RT: Room temperature
TRD: Tractional retinal detachment
TRITC: 5(6)-Tetramethylrhodaminisothiocyanate
VEGF: Vascular endothelial growth factor
VH: Vitreous hemorrhage
VRI: Vitreoretinal interface

## References

[1] Yau JWY, Rogers SL, Kawasaki R, Lamoureux EL, Kowalski JW, Bek T, et al. Global prevalence and major risk factors of diabetic retinopathy. Diabetes Care. 2012;35:556–64. 22301125 10.2337/dc11-1909 PMC3322721

[2] Saaddine JB. Projection of diabetic retinopathy and other major eye diseases among people with diabetes mellitus: United States, 2005–2050. Arch Ophthalmol. 2008;126:1740. 19064858 10.1001/archopht.126.12.1740

[3] Stitt AW, Curtis TM, Chen M, Medina RJ, McKay GJ, Jenkins A, et al. The progress in understanding and treatment of diabetic retinopathy. Prog Retin Eye Res. 2016;51:156–86. 26297071 10.1016/j.preteyeres.2015.08.001

[4] Agarwal D, Gelman R, Prospero Ponce C, Stevenson W, Christoforidis JB. The vitreomacular interface in diabetic retinopathy. J Ophthalmol. 2015;2015:1–10. 10.1155/2015/392983 PMC4573635 26425349

[5] El Rami H, Barham R, Sun JK, Silva PS. Evidence-based treatment of diabetic retinopathy. Semin Ophthalmol. 2017;32:67–74. 27700224 10.1080/08820538.2016.1228397

[6] Wong TY, Haskova Z, Asik K, Baumal CR, Csaky KG, Eter N, et al. Faricimab treat-and-extend for diabetic macular edema. Ophthalmology. 2024;131:708–23. 38158159 10.1016/j.ophtha.2023.12.026

[7] Writing Committee for the Diabetic Retinopathy Clinical Research Network, Gross JG, Glassman AR, Jampol LM, Inusah S, Aiello LP, et al. Panretinal photocoagulation vs intravitreous ranibizumab for proliferative diabetic retinopathy: a randomized clinical trial. JAMA. 2015;314:2137. 26565927 10.1001/jama.2015.15217 PMC5567801

[8] Rush RB. One year results of faricimab for aflibercept-resistant diabetic macular edema. Clin Ophthalmol. 2023;17:2397–403. 37605765 10.2147/OPTH.S424314 PMC10440101

[9] Akiba J, Arzabe CW, Trempe CL. Posterior vitreous detachment and neovascularization in diabetic retinopathy. Ophthalmology. 1990;97:889–91. 2381702 10.1016/s0161-6420(90)32486-7

[10] Blankenship GW, Machemer R. Long-term diabetic vitrectomy results. Report of 10 year follow-up. Ophthalmology. 1985;92:503–6. 2582329 10.1016/s0161-6420(85)34015-0

[11] Boneva SK, Wolf J, Rosmus D-D, Schlecht A, Prinz G, Laich Y, et al. Transcriptional profiling uncovers human hyalocytes as a unique innate immune cell population. Front Immunol. 2020;11:567274. 33042148 10.3389/fimmu.2020.567274 PMC7517040

[12] Wolf J, Boneva S, Rosmus D-D, Agostini H, Schlunck G, Wieghofer P, et al. Deciphering the molecular signature of human hyalocytes in relation to other innate immune cell populations. Invest Ophthalmol Vis Sci. 2022;63:9. 35266958 10.1167/iovs.63.3.9 PMC8934546

[13] Qiao H, Hisatomi T, Sonoda K-H, Kura S, Sassa Y, Kinoshita S, et al. The characterisation of hyalocytes: the origin, phenotype, and turnover. Br J Ophthalmol. 2005;89:513–7. 15774935 10.1136/bjo.2004.050658 PMC1772586

[14] Boeck M, Thien A, Wolf J, Hagemeyer N, Laich Y, Yusuf D, et al. Temporospatial distribution and transcriptional profile of retinal microglia in the oxygen-induced retinopathy mouse model. Glia. 2020;68:1859–73. 32150307 10.1002/glia.23810

[15] Wieghofer P, Hagemeyer N, Sankowski R, Schlecht A, Staszewski O, Amann L, et al. Mapping the origin and fate of myeloid cells in distinct compartments of the eye by single-cell profiling. EMBO J. 2021;40:e105123. 33555074 10.15252/embj.2020105123 PMC7957431

[16] Boneva SK, Wolf J, Hajdú RI, Prinz G, Salié H, Schlecht A, et al. In-depth molecular characterization of neovascular membranes suggests a role for hyalocyte-to-myofibroblast transdifferentiation in proliferative diabetic retinopathy. Front Immunol. 2021;12:757607. 34795670 10.3389/fimmu.2021.757607 PMC8593213

[17] Jones CH, Gui W, Schumann RG, Boneva SK, Lange CAK, Van Overdam KA, et al. Hyalocytes in proliferative vitreo-retinal diseases. Expert Rev Ophthalmol. 2022;17:263–80. 36466118 10.1080/17469899.2022.2100764 PMC9718005

[18] Migacz JV, Otero-Marquez O, Zhou R, Rickford K, Murillo B, Zhou DB, et al. Imaging of vitreous cortex hyalocyte dynamics using non-confocal quadrant-detection adaptive optics scanning light ophthalmoscopy in human subjects. Biomed Opt Express. 2022;13:1755. 35414987 10.1364/BOE.449417 PMC8973177

[19] Jalili V, Afgan E, Gu Q, Clements D, Blankenberg D, Goecks J, et al. The Galaxy platform for accessible, reproducible and collaborative biomedical analyses: 2020 update. Nucleic Acids Res. 2020;48:W395–402. 32479607 10.1093/nar/gkaa434 PMC7319590

[20] Wolf J, Boneva S, Schlecht A, Lapp T, Auw-Haedrich C, Lagrèze W, et al. The Human Eye Transcriptome Atlas: a searchable comparative transcriptome database for healthy and diseased human eye tissue. Genomics. 2022;114:110286. 35124170 10.1016/j.ygeno.2022.110286

[21] Schnichels S, Paquet-Durand F, Löscher M, Tsai T, Hurst J, Joachim SC, et al. Retina in a dish: cell cultures, retinal explants and animal models for common diseases of the retina. Prog Retin Eye Res. 2021;81:100880. 32721458 10.1016/j.preteyeres.2020.100880

[22] Bayik D, Tross D, Haile LA, Verthelyi D, Klinman DM. Regulation of the maturation of human monocytes into immunosuppressive macrophages. Blood Adv. 2017;1:2510–9. 29296902 10.1182/bloodadvances.2017011221 PMC5728638

[23] Zwadlo G, Bröcker EB, von Bassewitz DB, Feige U, Sorg C. A monoclonal antibody to a differentiation antigen present on mature human macrophages and absent from monocytes. J Immunol. 1985;134:1487–92. 3881524

[24] Joussen AM, Poulaki V, Le ML, Koizumi K, Esser C, Janicki H, et al. A central role for inflammation in the pathogenesis of diabetic retinopathy. FASEB J. 2004;18:1450–2. 15231732 10.1096/fj.03-1476fje

[25] Delaby C, Pilard N, Puy H, Canonne-Hergaux F. Sequential regulation of ferroportin expression after erythrophagocytosis in murine macrophages: early mRNA induction by haem, followed by iron-dependent protein expression. Biochem J. 2008;411:123–31. 18072938 10.1042/BJ20071474

[26] Pfefferlé M, Ingoglia G, Schaer CA, Yalamanoglu A, Buzzi R, Dubach IL, et al. Hemolysis transforms liver macrophages into antiinflammatory erythrophagocytes. J Clin Invest. 2020;130:5576–90. 32663195 10.1172/JCI137282 PMC7524492

[27] Nairz M, Theurl I, Swirski FK, Weiss G. Pumping iron-how macrophages handle iron at the systemic, microenvironmental, and cellular levels. Pflugers Arch. 2017;469:397–418. 28251312 10.1007/s00424-017-1944-8 PMC5362662

[28] Bressler SB, Beaulieu WT, Glassman AR, Gross JG, Jampol LM, Melia M, et al. Factors associated with worsening proliferative diabetic retinopathy in eyes treated with panretinal photocoagulation or ranibizumab. Ophthalmology. 2017;124:431–9. 28161147 10.1016/j.ophtha.2016.12.005 PMC6648671

[29] Lange CAK, Stavrakas P, Luhmann UFO, De Silva DJ, Ali RR, Gregor ZJ, et al. Intraocular oxygen distribution in advanced proliferative diabetic retinopathy. Am J Ophthalmol. 2011;152:406–e4123. 21723532 10.1016/j.ajo.2011.02.014

[30] Yao Y, Li R, Du J, Long L, Li X, Luo N. Interleukin-6 and diabetic retinopathy: a systematic review and meta-analysis. Curr Eye Res. 2019;44:564–74. 30644770 10.1080/02713683.2019.1570274

[31] Sharma S. Interleukin-6 trans-signaling: a pathway with therapeutic potential for diabetic retinopathy. Front Physiol. 2021;12:689429. 34093244 10.3389/fphys.2021.689429 PMC8170152

[32] Agostini H, Abreu F, Baumal CR, Chang DS, G. Csaky K, Demetriades AM, et al. Faricimab for neovascular age-related macular degeneration and diabetic macular edema: From preclinical studies to phase 3 outcomes. Graefes Arch Clin Exp Ophthalmol. 2024;online ahead of print. 10.1007/s00417-024-06531-9 PMC11584429 38847896

[33] Watanabe D, Suzuma K, Suzuma I, Ohashi H, Ojima T, Kurimoto M, et al. Vitreous levels of angiopoietin 2 and vascular endothelial growth factor in patients with proliferative diabetic retinopathy. Am J Ophthalmol. 2005;139:476–81. 15767056 10.1016/j.ajo.2004.10.004

[34] Loporchio DF, Tam EK, Cho J, Chung J, Jun GR, Xia W, et al. Cytokine levels in human vitreous in proliferative diabetic retinopathy. Cells. 2021;10:1069. 33946446 10.3390/cells10051069 PMC8147162

[35] Wu F, Phone A, Lamy R, Ma D, Laotaweerungsawat S, Chen Y, et al. Correlation of aqueous, vitreous, and plasma cytokine levels in patients with proliferative diabetic retinopathy. Invest Ophthalmol Vis Sci. 2020;61:26. 32084272 10.1167/iovs.61.2.26 PMC7326572

[36] Wolf J, Rasmussen DK, Sun YJ, Vu JT, Wang E, Espinosa C, et al. Liquid-biopsy proteomics combined with AI identifies cellular drivers of eye aging and disease *in vivo*. Cell. 2023;186:4868–e488412. 37863056 10.1016/j.cell.2023.09.012 PMC10720485

[37] Abd-Elrahman I, Meir K, Kosuge H, Ben-Nun Y, Weiss Sadan T, Rubinstein C, et al. Characterizing cathepsin activity and macrophage subtypes in excised human carotid plaques. Stroke. 2016;47:1101–8. 26941255 10.1161/STROKEAHA.115.011573

[38] Hannaford J, Guo H, Chen X. Involvement of cathepsins B and L in inflammation and cholesterol trafficking protein NPC2 secretion in macrophages. Obesity. 2013;21:1586–95. 23666609 10.1002/oby.20136 PMC6445554

[39] Hausmann M, Obermeier F, Schreiter K, Spottl T, Falk W, Schölmerich J, et al. Cathepsin D is up-regulated in inflammatory bowel disease macrophages. Clin Exp Immunol. 2004;136:157–67. 15030527 10.1111/j.1365-2249.2004.02420.x PMC1808992

[40] Schlecht A, Boneva S, Gruber M, Zhang P, Horres R, Bucher F, et al. Transcriptomic characterization of human choroidal neovascular membranes identifies calprotectin as a novel biomarker for patients with age-related macular degeneration. Am J Pathol. 2020;190:1632–42. 32339498 10.1016/j.ajpath.2020.04.004

[41] Wang S, Song R, Wang Z, Jing Z, Wang S, Ma J. S100A8/A9 in inflammation. Front Immunol. 2018;9:1298. 29942307 10.3389/fimmu.2018.01298 PMC6004386

[42] Cesaro A, Anceriz N, Plante A, Pagé N, Tardif MR, Tessier PA. An inflammation loop orchestrated by S100A9 and calprotectin is critical for development of arthritis. Bobé P. Editor PLoS ONE. 2012;7:e45478. 10.1371/journal.pone.0045478 PMC3445527 23029038

[43] Fan Y, Zhang J, Cai L, Wang S, Liu C, Zhang Y, et al. The effect of anti-inflammatory properties of ferritin light chain on lipopolysaccharide-induced inflammatory response in murine macrophages. Biochim Biophys Acta. 2014;1843:2775–83. 24983770 10.1016/j.bbamcr.2014.06.015

[44] Ciudin A, Hernández C, Simó R. Iron overload in diabetic retinopathy: a cause or a consequence of impaired mechanisms? Exp Diabetes Res. 2010;2010:714108. 20827392 10.1155/2010/714108 PMC2935195

[45] Boneva SK, Wolf J, Wieghofer P, Sebag J, Lange CA. Hyalocyte functions and immunology. Expert Rev Ophthalmol. 2022;17:249–62.

[46] Hamburg A. Some investigations on the cells of the vitreous body. Ophthalmologica. 1959;138:81–107. 14399214 10.1159/000303618

[47] Knutson M, Wessling-Resnick M. Iron metabolism in the reticuloendothelial system. Crit Rev Biochem Mol Biol. 2003;38:61–88. 12641343 10.1080/713609210

[48] Oldenborg P-A, Zheleznyak A, Fang Y-F, Lagenaur CF, Gresham HD, Lindberg FP. Role of CD47 as a marker of self on red blood cells. Science. 2000;288:2051–4. 10856220 10.1126/science.288.5473.2051

[49] Kelley SM, Ravichandran KS. Putting the brakes on phagocytosis: don’t-eat‐me signaling in physiology and disease. EMBO Rep. 2021;22:e52564. 34041845 10.15252/embr.202152564 PMC8183410

[50] Schulz A, Wahl S, Rickmann A, Ludwig J, Stanzel BV, Von Briesen H, et al. Age-related loss of human vitreal viscoelasticity. Trans Vis Sci Tech. 2019;8:56. 10.1167/tvst.8.3.56 PMC6602139 31293811

