# Supplementary figures and images for: The multifaceted role of vitreous hyalocytes: Orchestrating inflammation, angiomodulation and erythrophagocytosis in proliferative diabetic retinopathy
Source: J Neuroinflammation. 2024 Nov 14;21:297. doi: 10.1186/s12974-024-03291-5 (PMC11566480; doi:10.1186/s12974-024-03291-5)

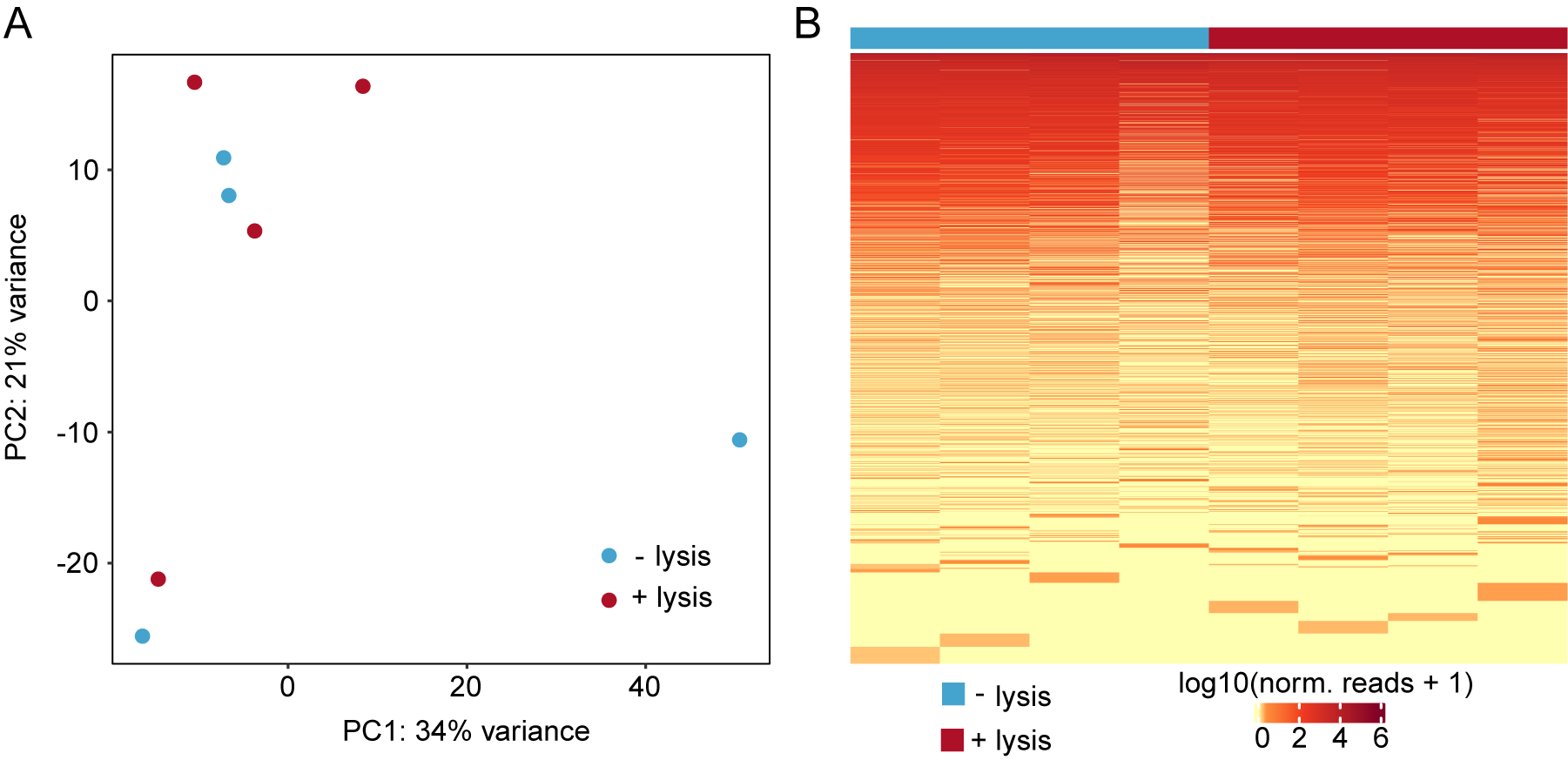

Supplement: Supplementary file 2 — Additional File 2 (Figure,.tif). Impact of Red Blood Cell (RBC) Lysis on hyalocyte expression. In order to assess the effects of the RBC lysis procedure on the transcriptional profile of hyalocytes, we conducted a preliminary analysis of control samples (each pooled from the vitreous tissue of 3 to 4 patients, see Table 1), which were processed to equal parts for a treatment with and without lysis. (A) Principal Component Analysis (PCA) demonstrating distribution of the analyzed entities: samples processed with lysis (“+ lysis”, dark red dots) and non-lysed samples (“- lysis”, light blue dots). The only sample in the right part of the graph was designated as a relative outlier, as less mapped reads were assigned to this sample than to other analyzed samples. (B) Unsupervised heatmap of expressed genes sorted according to mean expression in all samples [file 12974_2024_3291_MOESM2_ESM.tif]

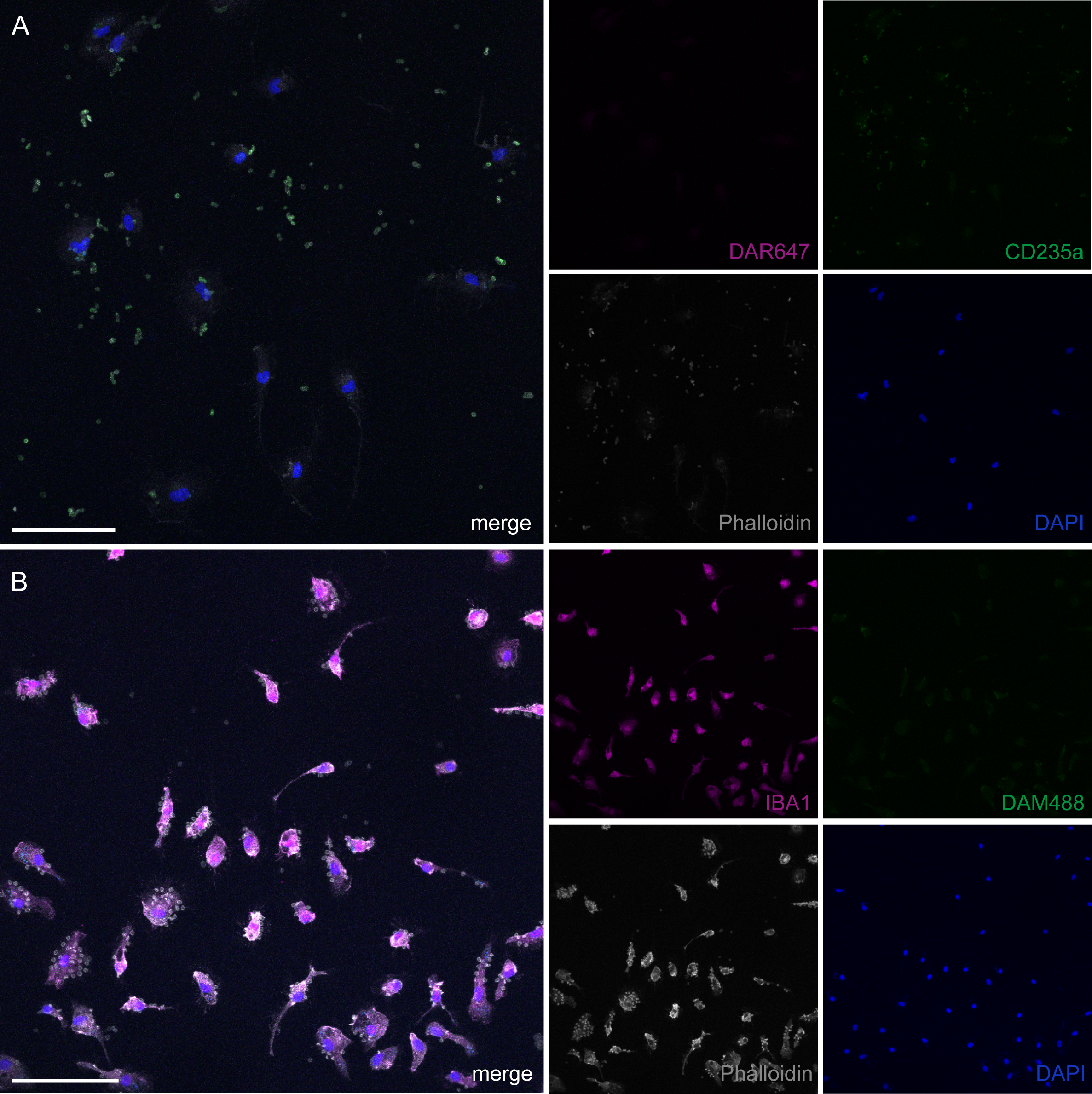

Supplement: Supplementary file 3 — Additional File 3 (Figure,.tif). Negative controls for immunohistochemistry. For negative controls, primary antibodies were omitted. Negative control for ionized calcium-binding adaptor molecule 1 (IBA-1, A) and cluster of differentiation 235a (CD235a) immunohistochemical staining (B) shown in Fig. 5B. Nuclei are counterstained with DAPI (4′,6-Diamidin-2-phenylindol). DAR647, donkey anti-rabbit Alexa Fluor 647. DAM488, donkey anti-mouse Alexa Fluor 488. Scale bars correspond to 100 μm [file 12974_2024_3291_MOESM3_ESM.tif]
